# Introducing a Chemically Intuitive Core-Substituent Fingerprint Designed to Explore Structural Requirements for Effective Similarity Searching and Machine Learning

**DOI:** 10.3390/molecules27072331

**Published:** 2022-04-04

**Authors:** Tiago Janela, Kosuke Takeuchi, Jürgen Bajorath

**Affiliations:** Department of Life Science Informatics and Data Science, B-IT, LIMES Program Unit Chemical Biology and Medicinal Chemistry, Rheinische Friedrich-Wilhelms-Universität, Friedrich-Hirzebruch-Allee 6, D-53115 Bonn, Germany; janela@bit.uni-bonn.de (T.J.); takeuchi@bit.uni-bonn.de (K.T.)

**Keywords:** molecular fingerprints, structural features, similarity searching, compound classification, machine learning

## Abstract

Fingerprint (FP) representations of chemical structure continue to be one of the most widely used types of molecular descriptors in chemoinformatics and computational medicinal chemistry. One often distinguishes between two- and three-dimensional (2D and 3D) FPs depending on whether they are derived from molecular graphs or conformations, respectively. Primary application areas for FPs include similarity searching and compound classification via machine learning, especially for hit identification. For these applications, 2D FPs are particularly popular, given their robustness and for the most part comparable (or better) performance to 3D FPs. While a variety of FP prototypes has been designed and evaluated during earlier times of chemoinformatics research, new developments have been rare over the past decade. At least in part, this has been due to the situation that topological (atom environment) FPs derived from molecular graphs have evolved as a gold standard in the field. We were interested in exploring the question of whether the amount of structural information captured by state-of-the-art 2D FPs is indeed required for effective similarity searching and compound classification or whether accounting for fewer structural features might be sufficient. Therefore, pursuing a “structural minimalist” approach, we designed and implemented a new 2D FP based upon ring and substituent fragments obtained by systematically decomposing large numbers of compounds from medicinal chemistry. The resulting FP termed core-substituent FP (CSFP) captures much smaller numbers of structural features than state-of-the-art 2D FPs. However, CSFP achieves high performance in similarity searching and machine learning, demonstrating that less structural information is required for establishing molecular similarity relationships than is often believed. Given its high performance and chemical tangibility, CSFP is also relevant for practical applications in medicinal chemistry.

## 1. Introduction

Similarity searching using molecular fingerprints (FPs) has a long history in chemoinformatics, especially for computational hit identification [1,2,3,4,5,6,7]. In similarity searching, the chemical similarity between query and database compounds is quantified on the basis of FP overlap and used to infer property similarity (such as a similar biological activity) [3,4,5]. FPs include bit string representations of chemical structures and/or molecular properties, as well as feature sets [1,2,3]. Various FP designs have been introduced to encode three-dimensional (3D) molecular features such as pharmacophore patterns or two-dimensional (2D) features such as substructures [1,5,8,9,10]. In addition, the bit string FP format has also been used to encode protein–ligand interactions [11,12]. By design, 2D FPs are simpler than 3D FPs but by no means less effective in detecting molecular similarity relationships and identifying new active compounds (2D FPs are typically less sensitive to feature noise than 3D FPs) [1,2,3,4,5]. Despite their conceptual simplicity, 2D FPs have often been surprisingly successful in identifying structurally diverse compounds with desired biological activities [2,3,4].

In general, 2D FPs include both keyed and hashed formats [2,3]. In keyed representations, each bit position corresponds to the presence or absence of a specific feature (or, albeit much less frequently used, a feature count). In hashed FPs, features are mapped to overlapping bit segments (hence producing specific bit patterns without 1:1 bit-to-feature correspondence). Accordingly, a bit position might be set on by different features (a phenomenon referred to as bit collision). Hashing algorithms can also be applied to transform molecule-specific feature sets into a bit string format of constant length. Furthermore, 2D FPs can be classified into two major and a few minor categories. The two major categories account for substructure-based FPs and others capturing topological patterns, as further discussed below. In addition, FPs have been introduced to encode 2D pharmacophore patterns [13,14], values of numerical 2D property descriptors [15], or combinations of binarized numerical descriptors and structural fragments [15,16]. 

For the major class of substructure-based FPs, molecular access system (MACCS) structural keys have been a pioneering prototype [17,18]. As the name implies, the design of these FPs is keyed and their predefined substructures include structural fragments and rule-based structural (SMARTS) patterns [18]. MACCS FP versions including 960 or 166 structural keys have been introduced, but the smaller 166-bit version has often met or even exceeded the performance of the larger one in similarity searching and has become a standard keyed FP design. Structural redundancy in such FPs often increases the noise in similarity calculations, especially for chemically complex molecules, and rational bit reduction strategies [16] or even random bit removal [19] have been applied to stabilize or further increase search performance. Other substructure-based FPs include variably sized versions of BCI FPs based upon a catalog of 1052 fragments [20] and the PubChem FP comprising 881 structural keys [21]. In addition, different types of atom pair-based FPs have been introduced [22,23,24]. However, none of these FP designs has replaced MACCS keys as a standard for substructure-based similarity searching. 

The other major class of 2D FPs comprises FPs capturing different types of topological patterns. One pioneering development has been the suite of daylight FPs that represent hashed designs with a length of up to 2048 bits and account for connectivity pathways through molecules [25]. The second class of topological FPs conceptually originates from the Morgan FP [26], another pioneering development, and includes various circular atom environment FPs [27,28,29]. Among these, extended connectivity FPs (ECFPs) capturing layered atom environments of varying bond diameters (e.g., four; corresponding to ECFP4) [29] have become a standard in the field, due to their typically highest search performance in 2D FP benchmarking [3,5,7]. ECFPs are directly based upon the Morgan algorithm and represent molecule-specific sets of layered atom environments. However, they are mostly used as constantly sized keyed or hashed bit strings, with ECFP4 being the most widely applied representative. In addition, ECFP variants with feature counts or feature groupings according to similar atom functions have been introduced [29] but are much less frequently used than ECFP4. Since many layered atom environments in compounds overlap, a characteristic of ECFPs is intrinsic topological feature redundancy, which (different from redundancy in some substructure FPs) does not notably affect search performance. Given its typically high performance, ECFP4 has become a gold standard for 2D similarity searching and is currently the most widely used FP. Over the past decade, with ECFPs becoming a mainstay in the field, conceptually new designs of FPs have been rare. 

In this study, we report a new 2D FP with lower feature numbers and bit density than commonly used FPs. This new FP was specifically designed to explore the question of how much structural information might be required for effective similarity searching. Exploring this question was inspired by earlier attempts to generate “mini-fingerprints” through reductionist approaches [16], as well as observations that, for given compound classes, even small sets of substructures from randomly generated populations might yield predictive FPs [30]. While dependent on specific classes of active compounds, these earlier observations partly inspired our current study, aiming to obtain further general insights into structural requirements for similarity searching. The new FP reported herein, termed core-substituent FP (CSFP), is keyed and particularly intuitive from a medicinal chemistry perspective. In addition to serving as a research tool for similarity searching and machine learning, the performance of CSFP compared with MACCS and ECFP4 observed in our study also indicates its potential for practical applications.

## 2. Results and Discussion

### 2.1. Fingerprint Design Principles

**Figure 1:**
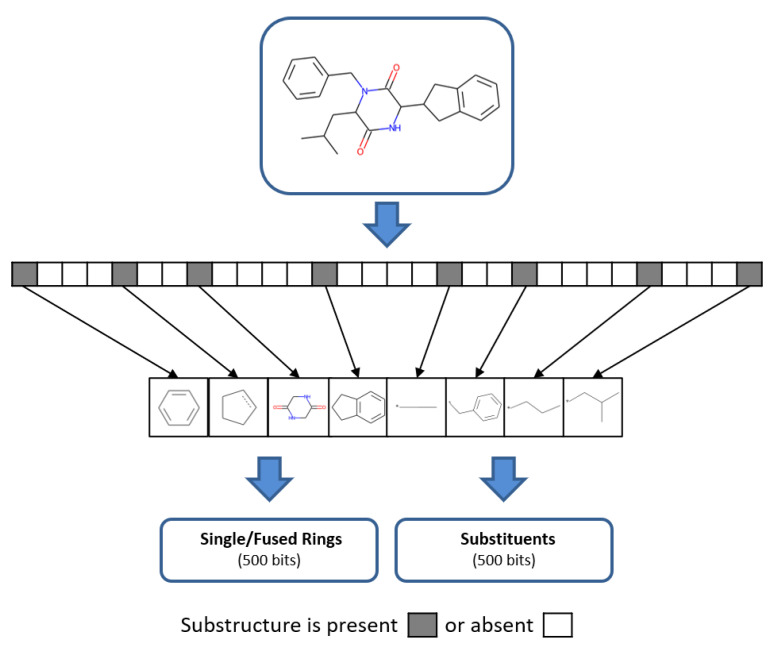
Keyed FP structure. The design of CSFP comprising a total of 1000 bit positions is schematically illustrated. Each bit position accounts for the presence or absence of a specific structural fragment. Ring fragments represent half of the bit positions and substituents the other half. Bit positions are set on (set to 1, gray) if the substructure is present in a molecule, or set off (white) if it is absent.

The general idea underlying the design of CSFP was the generation of an easily interpretable FP consisting of molecular fragments for representing as many compounds as possible with as little structural information per compound as possible. Hence, following a “structural minimalist” approach, as reflected by the number of structural features detected in a compound, a keyed substructure FP was envisioned comprising ring fragments from compound cores plus substituents, as illustrated in Figure 1, representing a new FP design strategy. For a given compound, we aimed to obtain fewer decomposed fragments than structural keys or atom environments, hence yielding a generally applicable FP with a smaller number of features than MACCS or ECFP4. Therefore, as a basis for CSFP design, a new fragment library was generated.

### 2.2. Molecular Fragments

#### 2.2.1. Fragment Categories

For our analysis, we distinguished between core and substituent fragments. For the generation of core fragments, analogue series (ASs) were algorithmically extracted isolated from compounds from medicinal chemistry sources, as specified in the Materials and Methods Section, and their core structures were sampled. Obtaining core structures from ASs ensured that the cores were represented by multiple compounds and were thus generalizable. All extracted cores contained ring structures, and these cores were then decomposed into fused and single rings, as described below. The resulting core fragments were complemented with an equally sized set of most frequently occurring substituent fragments selected from an R-group replacement database we have recently generated and made publicly available [31], resulting from a new methodology for systematically extracting R-groups from compounds [32].

#### 2.2.2. Core Structure Fragmentation

**Figure 2:**
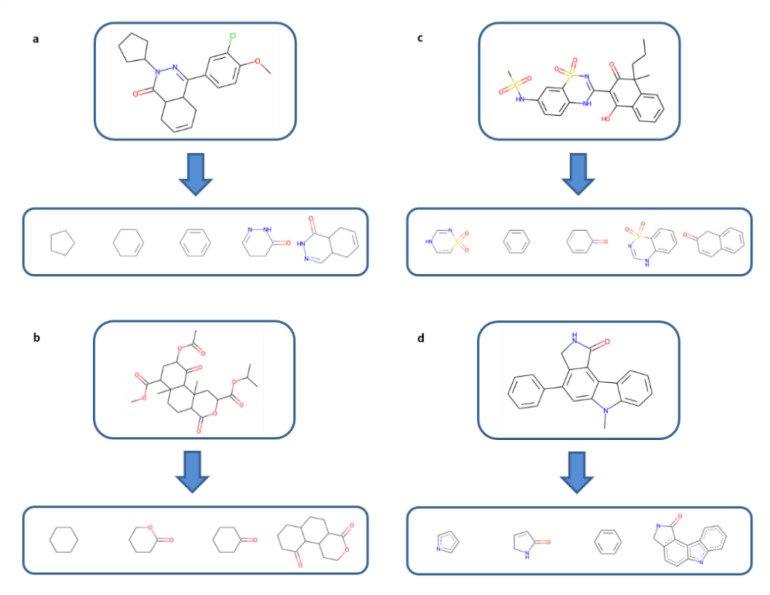
Generation of ring fragments: (**a**,**b**) illustrate the generation of chemically intact rings, and (**c**,**d**) the extraction of ring fragments from fused rings with retained hybridization states, making it possible to detect such model fragments in complex ring systems of test compounds.

Accounting only for the core structure of a compound would produce a single feature, which is of course not suitable for FP design. Therefore, from core structures, all fused and single rings were extracted first by the removal of single bonds between ring systems. Subsequently, fused rings were decomposed into individual ring fragments. All single and fused rings extracted from each core were recorded. Figure 2a–d illustrate the systematic extraction of fused and single rings from cores of exemplary compounds belonging to different ASs (in these cases, removal of all single-bonded substituents from rings yields the core). Importantly, for ring fragments extracted from fused rings, atomic hybridization states were retained such that the rings fragments—once encoded in an FP—were capable of matching individual rings in larger rings systems, especially aromatic rings. Hence, obtained single rings included chemically intact rings, as well as model ring fragments with hybridization states not existing in isolation, representing a special ring feature introduced for the design of CSFP.

#### 2.2.3. Ring and Substituent Fragments

From the ChEMBL database (version 28) [33], compounds were selected with reported high-confidence activity data for human targets, yielding a total of 67,165 unique active compounds. From these compounds, 7728 unique cores containing at least one ring were obtained. These cores yielded 1116 unique fused and 542 single ring fragments. 

The R-group replacement database was also extracted from ChEMBL compounds [31]. The benzene ring and H atom were excluded as substituents. Accordingly, the benzene ring was only permitted as a ring fragment, thereby avoiding ambiguous assignments that would occur with high frequency. From the R-group resource, the 500 most frequent substituents obtained by random bond fragmentation and by fragmentation on the basis of retrosynthetic rules [32], respectively, were selected and combined. These subsets displayed a large overlap, as to be expected, and yielded a total of 661 unique substituent fragments.

### 2.3. Fingerprint Assembly and Feature Mapping

From the isolated ring fragments, the 250 single and the 250 fused rings that most frequently occurred in active compounds from medicinal chemistry were selected and combined with the 500 most frequent R-groups. These fragments were combined to generate CSFP comprising a total of 1000 bits with a balanced composition of rings and substituents, with each fragment assigned to a single bit position, representing a prototypic keyed design.

To ensure that test compounds produced bit patterns sufficient for meaningful similarity comparison, substructure relationships between fragments were considered. Hence, individual fused rings or substituents set multiple CSFP bits on if they contained other recorded rings or substituents as substructures, respectively, as illustrated in Figure 1. Hence, as mentioned above, if a fused ring recorded in CSFP was detected, its bit was set on as well as the bits of decomposition fragments, if available. Likewise, if a substituent was detected containing, for example, two others as substructures, three bits were set on (that is, one for the complete substituent and one for each substructure). 

### 2.4. Compound Activity Classes

To evaluate feature distributions in FPs and compare their performance, a set of 30 compound activity classes representing different degrees of difficulty for similarity searching was used. This set included 10 “easy” (structurally homogeneous) classes typically yielding accurate results in similarity searching, 10 “intermediate”, and 10 “difficult” (structurally heterogeneous) classes often yielding moderate or low prediction accuracy, as previously reported [34]. Since their original exploration and categorization, many additional compounds have become available for the selected activity classes, which we curated for our study (see Materials and Methods). These up-to-date versions of these activity classes covered diverse targets and contained between 121 and 3159 compounds. Supplementary Materials Table S1 reports the composition of all activity classes.

### 2.5. Feature Distribution

**Figure 3:**
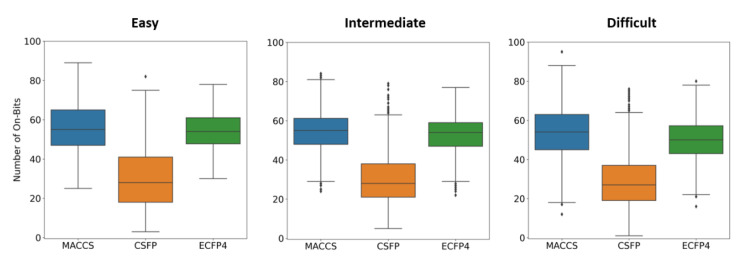
Distribution of FP features. Boxplots show the distribution of features (bits set on) for MACCS (blue), CSFP (gold), and ECFP4 (green) and a random sample of 1000 compounds from all activity classes. In boxplots, the upper and lower whiskers indicate maximum and minimum values, the boundaries of the box represent the upper and lower quartiles, values classified as statistical outliers are shown as diamonds, and the median value is indicated by a horizontal line.

**Figure 4:**
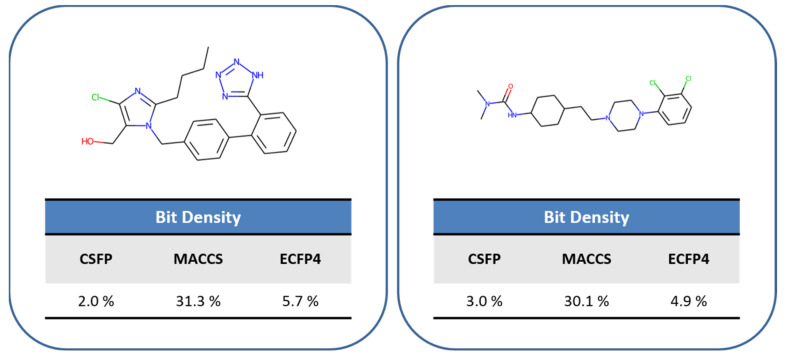
Comparison of FP bit density. For two exemplary compounds, the bit density (percentage of bits set on) of CSFP, MACCS, and ECFP4 is reported.

We first assessed the major goal of CSFP design, that is, producing a reference FP accounting for fewer structural features than the standard 166-bit version of MACCS and the 1024-bit version ECFP4. Therefore, the three FPs were calculated for all compound activity classes and the FP feature distributions were determined. Figure 3 compares these distributions, revealing that the CSFP design goal was fully met. Regardless of the activity class category, the feature distribution was very similar for each FP. However, the number of CSFP features was consistently much smaller than the number of MACCS or ECFP4 features. While MACCS and ECFPs captured a similar number of features, with median values of 55 and 53 features per FP over all combined activity classes, respectively, a corresponding median value of only 28 features per CSFP was observed. Figure 4 shows bit densities of the three FPs for two exemplary compounds, illustrating the lower bit density of CSFP, which was also generally observed. Supplementary Figures S1 and S2 from the Supplementary Materials show the most frequently detected CSFP ring and substituents fragments, respectively, in easy, intermediate, and difficult activity classes, revealing overall balanced distributions of CSFP fragments, with only a few prevalent rings or substituents (such as, for example, the pyridine ring or methyl and amino group). Taken together, these findings confirmed that CSFP captured overall a much lower number of features than the MACCS and ECFP4 standards.

### 2.6. Performance Evaluation

We then compared the performance of the three FPs in similarity searching and compound classification via machine learning. Since FPs are often used as descriptors in molecular machine learning, we carried out support vector machine (SVM) and random forest (RF) calculations to distinguish compounds from each activity class from a random sample of ChEMBL compounds. The results of similarity searching and compound classification were evaluated on the basis of different performance metrics (calculation setups and performance measures are detailed in Materials and Methods).

#### 2.6.1. Similarity Searching

**Figure 5:**
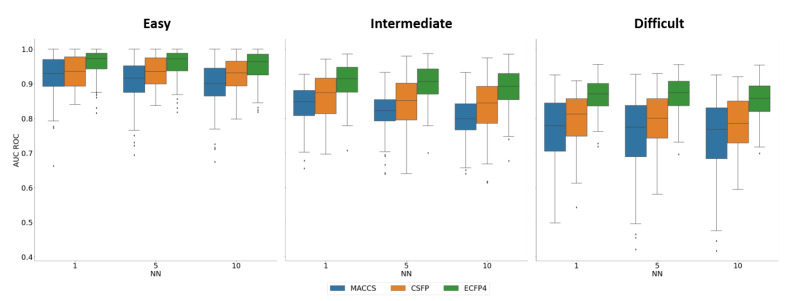
Similarity searching. Boxplots report the mean AUC ROC values for 1-, 5-, and 10-NN similarity searching with 10 reference compounds across all activity classes.

Figure 5 summarizes the results of our systematic similarity search calculations carried out with multiple reference compounds and 1-, 5-, and 10-nearest neighbor (NN) similarity assessment, respectively. As expected, similarity search performance generally decreased from easy over intermediate to difficult activity classes for all FPs, resulting in highly to moderately accurate compound rankings, as assessed on the basis of area under the receiver-operating characteristics curve (AUC ROC) values. From the distributions of obtained AUC ROC values, a clear picture emerged that ECFP4 generally performed best, followed by CSFP, and MACCs. With an increasing degree of difficulty, the performance gaps slightly widened, but the difference between AUR ROC values remained relatively small (mostly falling with a 0.1 value interval). Hence, despite the much lower number of structural features captured by CSFP, its similarity performance exceeded (MACCS) or approached (ECFP4) the accuracy of the standard FPs. All observed differences between AUR ROC distributions were statically significant (Wilcoxon test, *p*-values < 0.05).

#### 2.6.2. Compound Classification

**Figure 6:**
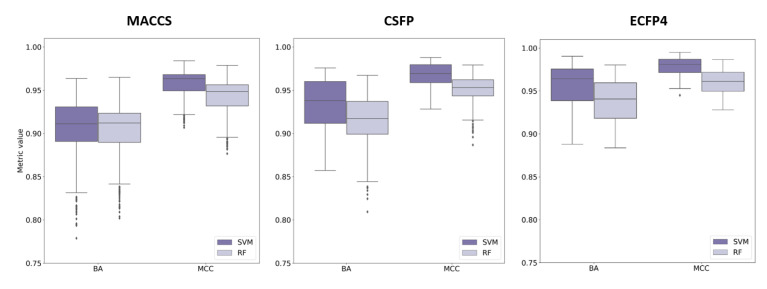
Compound classification. Boxplots report the performance of machine learning models (SVM: blue, RF: orange) using MACCS, CSFP, and ECFP4 in compound classification based on the BA and MCC measures.

Similar observations were made for machine learning models used for compound classification. Figure 6 summarizes SVM and RF results for all activity classes based upon balanced accuracy (BA) and the Matthews correlation coefficient (MCC) (Supplementary Figure S3 from the Supplementary Materials shows all results). SVM and RF classification accuracy was, overall, comparably high. For example, even for difficult classes, BA median values greater than 0.95 and MCC median values greater than 0.9 were observed, reflecting accurate calculations. Here, there were small advantages of ECFP4 over CSFP for some but not all performance measures (Figure S3 from the Supplementary Materials). Differences between MCC value distributions were statistically significant for at least 80% of the activity classes (Wilcoxon test, *p*-values < 0.05). Overall, however, SVM and RF classification accuracy achieved on the basis of CSFP and ECFP4 was comparable, thus corroborating the results obtained in systematic similarity searching.

## 3. Materials and Methods

### 3.1. Compound Activity Classes

From ChEMBL (version 28) [33], compounds with less than 1000 Da, a direct target annotation (target confidence score: 9), and an exact potency value (standard relation: “=”), given as K_i_, IC_50,_ or K_d_ values, were selected. Interactions with potency values of less than 10 µM or interactions flagged as “inactive”, “not active”, “inconclusive”, or “potential transcription error” were disregarded. After removing undesired targets, such as hERG, serum-albumin, or ABC transporters (i.e., pharmaceutical anti-targets, inhibition of which is undesired), compounds with potential assay interference characteristics were removed using publicly available filters [35,36,37].

Based on these criteria, 231,772 unique compounds were obtained with a total of 351,198 activity measurements for 1940 human targets and recorded as SMILES strings [38]. 

From these high-confidence data sets, a total of 30 compound activity classes (each class represents compounds with activity against a specific target) were selected for benchmarking falling into “easy”, “intermediate”, and “difficult” categories (10 classes per category) for similarity searching and compound classification, as discussed above. 

### 3.2. Core Generation and Fragmentation

ASs were systematically extracted from all ChEMBL compounds with available high-confidence activity data using the compound–core relationship (CCR) algorithm [39]. The CCR algorithm systematically fragments all combinations of 1–5 exocyclic bonds in a compound (applying a 2:1 core-to-substituent size ratio). From ASs, core structures were extracted and generalized by the addition of hydrogen atoms to all substitution sites [39]. Ring fragments were extracted from core structures using the protocol available in RDKit [35].

### 3.3. Molecular Representations

CSFP was compared with the 166-bit version of MACCS and the 1024-bit version of ECFP4 generated using RDKit.

### 3.4. Similarity Searching

For each similarity search trial, 100 active compounds were randomly selected from each activity class. Then, 10 of these compounds were randomly selected as reference compounds, and the remaining 90 active compounds were added as potential hits to a background database consisting of a random sample of 100,000 ChEMBL compounds (excluding each activity class). For each class, 20 independent trials were carried out, and the results were averaged.

In each trial, the *k*-nearest neighbor (k-NN) search strategy was applied [40] including 1-NN, 5-NN, and 10-NN calculations. The similarity scores were assessed by calculating the Tanimoto coefficient (Tc) [41]. For 1-NN, a database compound was compared with all 10 reference compounds, and the highest Tc value was selected as the final similarity score. In 5-NN and 10-NN calculations, the top 5 and 10 similarity values were averaged, respectively, to obtain the final similarity score for each database compound. 

### 3.5. Machine Learning

For machine learning calculations, random forest (RF) [42] and support vector machine (SVM) [43] were used. All RF and SVM models were implemented using scikit-learn [44].

#### 3.5.1. Random Forest

The RF is a supervised machine learning algorithm that derives an ensemble of decision trees generated from randomly selected training instances using bootstrapping. In predictions, each tree yields a class label for a test instance, and the final class label is determined by an ensemble majority vote [42].

#### 3.5.2. Support Vector Machine

SVM is a supervised learning method deriving a hyperplane in feature space that optimally separates training instances with different class labels by maximizing the separating margin of the hyperplane. If linear separation is not possible in the feature space, a kernel function is applied to project the training data into a higher-dimensional space where linear separation might become possible [43].

#### 3.5.3. Model Building and Hyperparameter Optimization

For each activity class, 50% of the compounds were randomly selected for training and hyperparameter optimization. The remaining 50% of the compounds served as an external validation set. For hyperparameter optimization, 10-fold internal cross-validation and grid search were applied. Optimal hyperparameters were chosen based on the mean balanced accuracy across all trials. 

For RF, the number of trees was determined by testing 25, 50, 100, 200, and 400 trees, and the minimum number of samples required to split an internal node by testing 2, 3, 5, and 10 samples. For all remaining hyperparameters, default settings were used.

For SVM, the cost hyperparameter C, which regulates the trade-off between misclassified samples and the margin size, was optimized using candidate values of 0.1, 1, 10, 50, 100, 200, and 1000. SVM models were derived using the Tanimoto kernel, a preferred choice for binary fingerprints [45]. Class weights were set to “balanced”. For all remaining hyperparameters, default settings were used.

#### 3.5.4. Predictions

RF and SVM models were applied to predict 50% of the compounds from each activity class not used for training (positive instances). As negative instances, three times the number of positive instances were randomly selected from ChEMBL (excluding each activity class). For each class, 20 independent trials were carried out, and the results of the predictions were averaged. 

### 3.6. Performance Measures

The performance of RF and SVM models was evaluated on the basis of different measures including balanced accuracy (BA) [46], Matthew’s correlation coefficient (MCC) [47], F1 score [48], precision, and recall. Similarity searching performance was evaluated according to the area under the ROC curve (AUC ROC) [49].

(1) $$\mathrm{BA}=\frac{1}{2}(\mathrm{TPR}+\mathrm{TNR})$$

(2) $$\mathrm{MCC}=\frac{\mathrm{TP}\times \mathrm{TN}-\mathrm{FP}\times \mathrm{FN}}{\sqrt{\left(\mathrm{TP}+\mathrm{FP})(\mathrm{TP}+\mathrm{FN})(\mathrm{TN}+\mathrm{FP})(\mathrm{TN}+\mathrm{FN}\right)}}$$

(3) $$F1=2\times \frac{\mathrm{TP}}{2\mathrm{TP}+\mathrm{FP}+\mathrm{FN}}$$

(4) $$\mathrm{precision}=\frac{\mathrm{TP}}{\mathrm{TP}+\mathrm{FP}}$$

(5) $$\mathrm{recall}=\frac{\mathrm{TP}}{\mathrm{TP}+\mathrm{FN}}$$

where TP, TN, FP, and FN stand for true positives, true negatives, false positives, and false negatives, respectively.

For results of similarity searching and compound classification, statistical significance assessment was based on AUC ROC and MCC values, respectively, using the nonparametric Wilcoxon test [50].

## 4. Conclusions

In this study, we have introduced a new generally applicable 2D FP designed to investigate the question of whether or not fewer structural features than commonly captured in state-of-the-art 2D FPs might be sufficient for correctly detecting molecular similarity relationships in similarity searching and compound classification. CSFP was assembled from ring and substituent fragments systematically extracted from biologically active compounds. A key aspect of its design is separately accounting for substructure relationships between rings and substituents, hence yielding multiple bit settings for fused rings and subsets of larger substituents and ensuring the presence of minimally required bit density for meaningful FP comparison. CSFP was shown to contain significantly fewer structural features than MACCS or ECFP4 but exceeded the predictive performance of MACCS in similarity searching and machine learning and approached (or met) the performance of ECFP4. Taken together, these findings demonstrated that a smaller number of FP features than that currently used is sufficient for the accurate detection of compound similarity relationships indicative of similar biological activity. Although CSFP was primarily designed as a research tool, its chemically intuitive nature and high-performance level also render it favorable for practical applications in medicinal chemistry. On the basis of the computational protocols provided herein and the substituent resource we have made publicly available, the CSFP design can be easily reproduced, modified, and further extended.

## Data Availability

All calculations were carried out using publicly available data.

## Supplementary Materials

The following supporting information can be downloaded at: https://www.mdpi.com/article/10.3390/molecules27072331/s1; Figure S1, Most frequent ring fragments, reports the CSFP frequency of ring fragments from easy, intermediate, and difficult activity classes, respectively. Figure S2, frequent substituent fragments, reports the CSFP frequency of substituent fragments from easy, intermediate, and difficult activity classes, respectively. Figure S3, Compound classification. Boxplots report RF and SVM results for MACCS, CSFP, and ECFP4 on the basis of different performance measures (see Materials and Methods) across all activity classes. Table S1. Compound activity classes. For each of the 30 activity classes used for our analysis, the ChEMBL target ID and the number of compounds are reported.

## Sample Availability

Samples from the compounds are not available from the authors upon request.
