# Supplementary material for: Introducing a Chemically Intuitive Core-Substituent Fingerprint Designed to Explore Structural Requirements for Effective Similarity Searching and Machine Learning
Source: Molecules. 2022 Apr 4;27(7):2331. doi: 10.3390/molecules27072331 (PMC9000322; doi:10.3390/molecules27072331)
Supplement: Supplementary file 1 [file molecules-27-02331-s001.zip › molecules-1658750-supplementary.pdf]

molecules

MDPI

*Supplementary Materials*

# Introducing a Chemically Intuitive Core-Substituent Fingerprint Designed to Explore Structural Requirements for Effective Similarity Searching and Machine Learning

**Tiago Janela, Kosuke Takeuchi and Jürgen Bajorath ***

Department of Life Science Informatics and Data Science, B-IT, LIMES Program Unit Chemical Biology and Medicinal Chemistry, Rheinische Friedrich-Wilhelms-Universität, Friedrich-Hirzebruch-Allee 6, D-53115 Bonn, Germany; janela@bit.uni-bonn.de (T.J.); takeuchi@bit.uni-bonn.de (K.T.)

* Correspondence: bajorath@bit.uni-bonn.de; Tel.: +49-228-7369-100

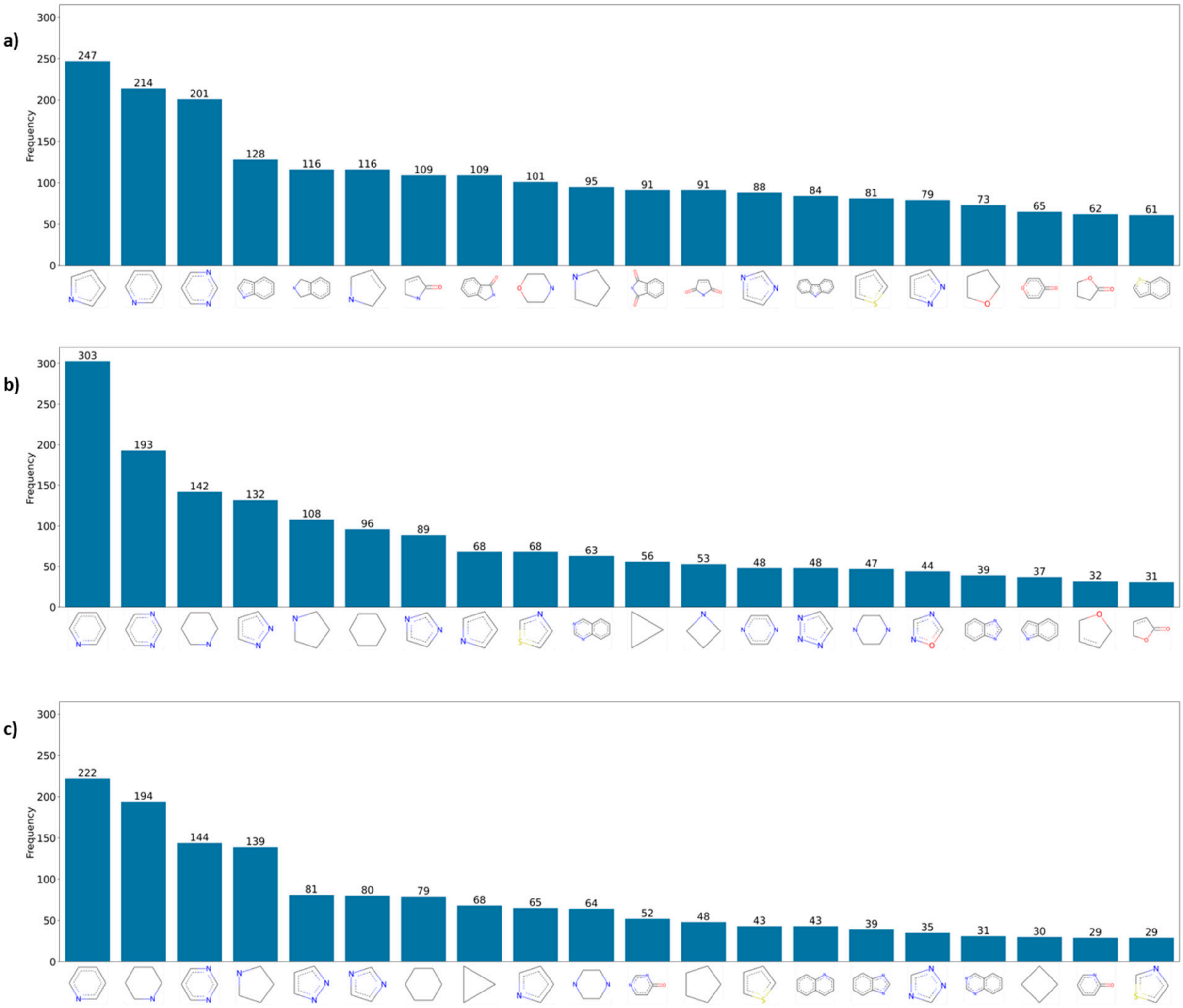


**Figure S1.** Most frequent ring fragments. (**a**), (**b**), and (**c**) report the CSFP frequency of ring fragments from easy, intermediate, and difficult activity classes, respectively.

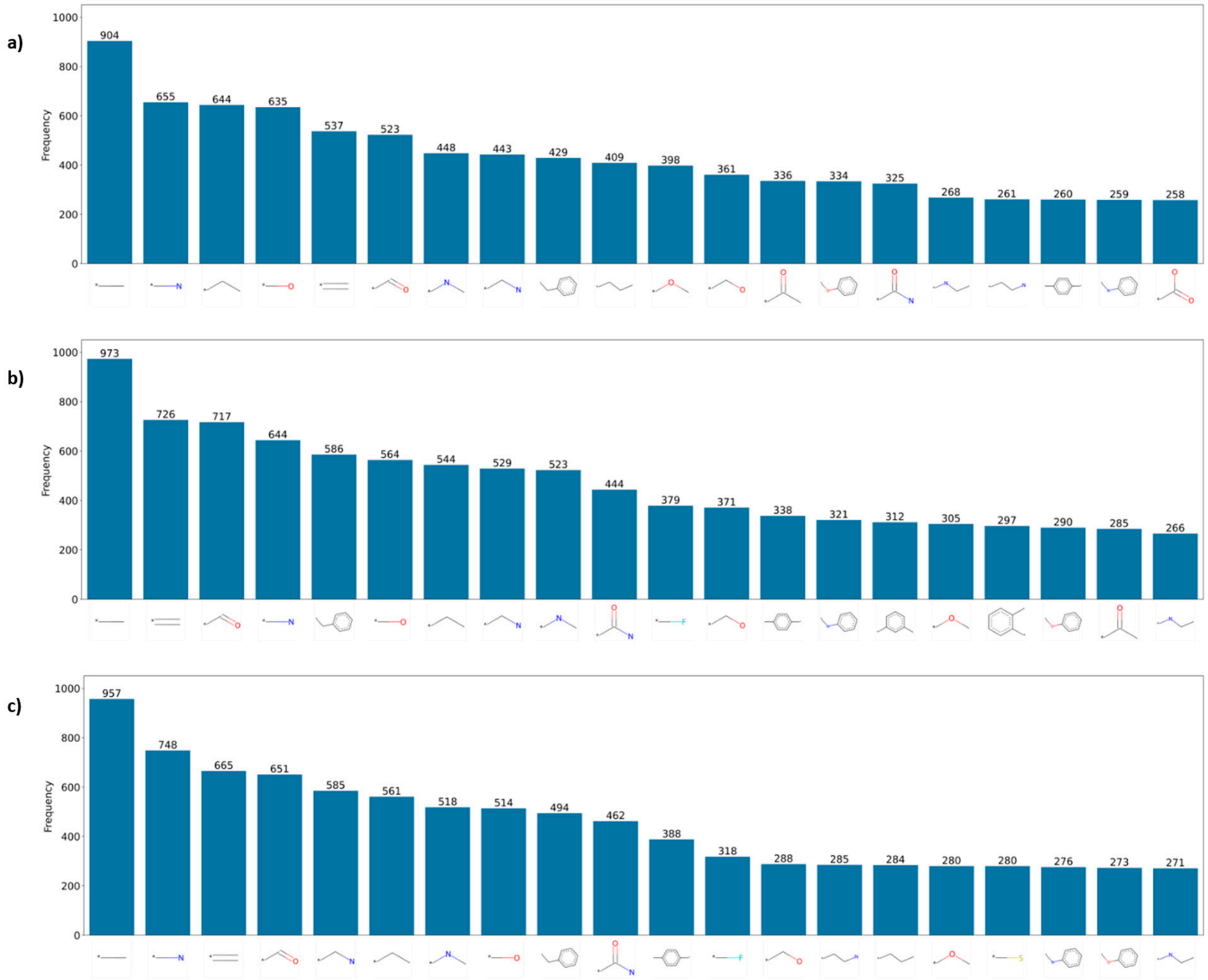


**Figure S2.** Most frequent substituent fragments. (**a**), (**b**), and (**c**) report the CSFP frequency of substituent fragments from easy, intermediate, and difficult activity classes, respectively.

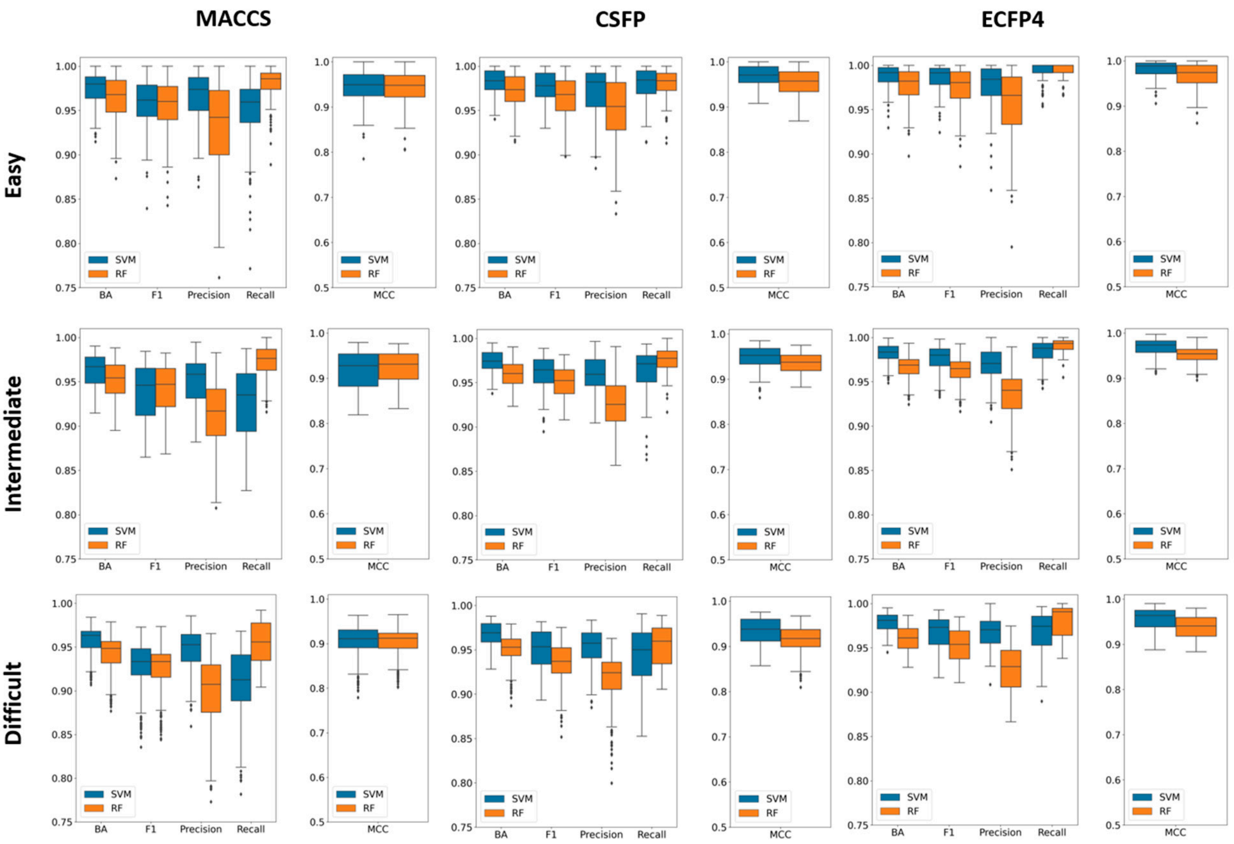


**Figure S3.** Compound classification. Boxplots report RF and SVM results for MACCS, CSFP, and ECFP4 on the basis of different performance measures (see Materials and Methods) across all activity classes.

**Table S1.** Compound activity classes. For each of the 30 activity classes used for our analysis, the ChEMBL target ID and the number of compounds are reported.

| Easy | | Intermediate | | Difficult | |
|---|---|---|---|---|---|
| **Target ID** | **# Compounds** | **Target ID** | **# Compounds** | **Target ID** | **# Compounds** |
| 2778 | 121 | 4618 | 261 | 4581 | 562 |
| 5491 | 145 | 4333 | 322 | 333 | 678 |
| 299 | 155 | 4792 | 473 | 261 | 1215 |
| 5443 | 176 | 4561 | 571 | 260 | 1300 |
| 2016 | 239 | 4616 | 641 | 4794 | 1939 |
| 4427 | 240 | 5145 | 965 | 279 | 1946 |
| 3142 | 249 | 4722 | 1382 | 344 | 2241 |
| 4198 | 253 | 5071 | 1513 | 284 | 2375 |
| 2695 | 473 | 4805 | 1561 | 264 | 2379 |
| 4550 | 1523 | 4822 | 2713 | 4235 | 2401 |
